# Digging for care-seeking behaviour among gold miners in the Guyana hinterland: a qualitative doer non-doer analysis of social and behavioural motivations for malaria testing and treatment

**DOI:** 10.1186/s12936-020-03289-3

**Published:** 2020-07-06

**Authors:** Shirley D. Yan, Jennifer Orkis, Saifra Khan Sohail, Sean Wilson, TrishAnn Davis, J. Douglas Storey

**Affiliations:** 1 Johns Hopkins Center for Communication Programs, 111 Market Place, Suite 310, Baltimore, MD USA; 2 Breakthrough ACTION Guyana, XX Barrack St., Georgetown, Guyana

**Keywords:** Guyana, Care-seeking, Gold miner, Social behaviour change, Malaria, Rapid diagnostic tests

## Abstract

**Background:**

Although Guyana has made significant progress toward malaria control, limited access to malaria testing and treatment services threatens those gains. Mining activities create breeding environments for mosquitoes, and the migrant and mobile mining populations are hard to reach with information and services. The Ministry of Public Health (MoPH) has trained volunteers to test and treat malaria cases in remote regions. However, it remains unclear how miners perceive these testers, the services they provide, or what their malaria care-seeking behaviour is in general. To better address these challenges, Breakthrough ACTION Guyana and MoPH conducted qualitative research from October to November 2018 in Regions 7 and 8 in Guyana.

**Methods:**

A total of 109 individuals, 70 miners, 17 other mining camp staff, and 22 other key stakeholders (e.g. community health workers, pharmacists, and regional leadership), participated in semi-structured interviews and focus group discussions. Results were derived using a framework analysis, with an adjusted doer and non-doer analysis, and organized using the integrated behaviour framework.

**Results:**

Miners sought MoPH-approved services because of close geographic proximity to testing services, a preference for public service treatment, and a desire to correctly diagnose and cure malaria rather than just treat its symptoms. Those who chose to initiate self-treatment—using unregulated medications from the private and informal sector—did so out of convenience and the belief that self-treatment had worked before. Miners who completed the full MoPH-approved treatment understood the need to complete the treatment, while those who prematurely stopped treatment did so because of medication side effects and a desire to feel better as soon as possible.

**Conclusion:**

Reasons why miners do and do not pursue malaria testing and treatment services are diverse. These results can inform better MoPH programming and new solutions to improve malaria outcomes in Guyana.

## Background

In South America, malaria affects less than one million people each year [1]. In Guyana, 13,936 total cases were reported in 2017, most of which were either *Plasmodium vivax* (54.9%) or *Plasmodium falciparum* (36.9%) (Trotman 2018, pers. commun.). Although the prevalence of malaria in South America is much smaller than the 192.6 million cases in Africa that account for approximately 90% of the total number of cases in the world, the resurgence of malaria in the last 30 years in South America is a growing public health concern; an exploration into this change can provide learnings for ongoing global malaria control [1, 2].

Challenges in Guyana include an increased prevalence of malaria in remote areas, potential increased drug-resistant parasites through unregulated medication use, and increased human activity from gold mining and logging industries in endemic areas [2, 3]. In Guyana, 70% of all recorded malaria cases are found in Regions 1, 7, 8, and 9. Malaria disproportionately affects men (70% of all cases), Amerindians (43% of all cases), and people aged 15 to 49 (73.2% of all cases) (Trotman 2018, pers. commun.).

Within these four regions, gold mining is a well-established industry that significantly contributes to the economy. In 2014, gold mining contributed USD 3.078 billion to Guyana’s gross domestic product and accounted for 43% of all exports in Guyana [4]. The Guiana Shield (which includes Guyana, Suriname, French Guiana, and parts of Venezuela and Brazil), is rich in minerals, including gold, bauxite, and diamonds, that attract both legal and illegal mining activities. Locally referred to as “pork-knockers,” deriving from miners’ appetite for pickled pork, miners in Guyana typically are from Guyana or neighbouring countries [5–7]. Miners often first meet at “landings” (typically road junctions or river crossings), which serve as hubs for miners to access the interior, get supplies, and socialize. These areas serve as resting places before or after mining activities and often take shape as either formal or informal towns. The formality of the landing depends on the economic activity around it: mining activities bring development in the form of roads, infrastructure, transportation, and businesses [8]. The mining industry serves as the economic backbone for landings, surrounding settlements, and towns; when mines close due to overmining, towns become deserted [6].

Across Guyana, Suriname, and French Guiana, malaria is most prevalent in the interior regions and is associated with mining populations [9–11]. Because of the high use of water for hydraulic mining operations and high deforestation rates, excavated pits, if not subsequently filled in with earth, fill with rainwater and become breeding grounds for mosquitoes [11, 12]. Deforested areas are more likely to have larval breeding sites because forest margins, rather than dense forests, provide optimal larval breeding grounds for *Anopheles darlingi* mosquitoes, which tend to bite indoors and at night across the Guiana Shield and Amazon [13–18]. In industries like gold mining and logging, malaria leads not only to poor health outcomes but also to economic loss for workers when they fall ill [9]. Given the high correlation between mining activity and malaria prevalence throughout South America and globally, mining populations and the mining industry play a crucial part to prevent and control of malaria [16, 19–23].

The introduction of unregulated medicines to treat malaria in Guyana and Suriname has played a role in the decreased efficacy of the recommended treatments [24]. Unregulated access and overuse of ineffective medications can lead to anti-malarial resistance because self-treatment, improper treatment regimens, and the use of substandard medications kill off more susceptible *Plasmodium*, which leaves *Plasmodium* that are anti-malarial resistant due to genetic mutations [25]. There have been documented cases of both chloroquine and primaquine resistant *P. vivax* malaria throughout South America, including Guyana [26].

In general, many malaria strategies and programmes focus on effective and low-cost prevention methods that reduce mosquito breeding or malaria transmission. In a study comparing insecticide-treated nets (ITNs), indoor residual spray (IRS), and use of artemisinin-based combination therapy (ACT) across all *P. falciparum* cases in sub-Saharan Africa from 2000 to 2015, ITNs contributed to 68% of cases averted, followed by 22% from ACT and 10% from IRS [27]. Recent studies also indicate that ITNs are more cost-effective. Published in 2011, a systematic review examined the cost-effectiveness of malaria control interventions and found that ITNs cost $2.20 per person, compared to the combined cost of diagnosis and treatment ($10.16/person) and IRS ($6.70/person) (these are global average costs, which may vary by location from distribution-specific costs) [28].

Additionally, in the summer of 2018, the National Malaria Programme (NMP) in Guyana conducted a mass distribution of long-lasting insecticide-treated net (LLIN) paired with an information, education, and communication (IEC) campaign targeting LLIN use throughout the endemic regions. This campaign distributed bed and hammock mosquito nets to Amerindian communities, mining camp managers, and other communities. Management of malaria requires focus and attention on high-quality diagnostics and treatment programmes as well. The World Health Organization (WHO) guidelines on malaria treatment advocate for early diagnosis and prompt effective treatment within 24 to 48 h of the onset of malaria symptoms, use of rapid diagnostic tests (RDTs) or microscopy for diagnosis, ACT for all episodes of malaria, full treatment adherence, and limiting unnecessary use of anti-malarial drugs (which could lead to anti-malarial resistance) [1]. Guyana’s malaria control efforts led to a 50% reduction in malaria cases from 2000 to 2014 [29]. The NMP aims to reduce malaria prevalence again by 50%, compared to 2014 rates, by 2020 [30].

To expand access to testing and treatment in the most affected regions of the country, the Ministry of Public Health (MoPH), Pan American Health Organization (PAHO), and Global Fund to Fight AIDS, Tuberculosis, and Malaria introduced free RDTs and treatment programme for mining camps and surrounding communities in endemic regions. Table 1 outlines malaria testing and treatment regimens available in public health facilities and programmes in Guyana. Malaria is easily mistaken for other diseases or can be asymptomatic; as a result, proper diagnosis is important to determine if patients have malaria and, if so, treat the appropriate malaria strain to protect against potential drug-resistance to reduce unnecessary wastage of medications [31]. RDTs are more cost-effective than microscopy, especially in rural areas, because they do not require as much capital cost, staff time, technical training, or supplies [32, 33]. For Guyana’s RDT programme, regional malaria staff trained cooks, security guards, camp managers, and shop owners (stable populations who are less likely to migrate) in and around mining camps to screen patients for malaria symptoms, use RDTs, administer appropriate medication given positive or negative test results, and fill out reporting forms [34]. Regional malaria staff assemble medication sachets for different malaria strains, with clear dosage instructions, so these testers can select the appropriate sachet for a parasite strain. Piloted in Region 8, and now implemented in Regions 1, 7, and 9, this program provides community-based and strain-specific care closer to the population in need, and reduces associated care-seeking barriers. This study aims to understand behavioural barriers and opportunities to improve testing and treatment for malaria as part of the first stage of the larger Breakthrough ACTION Guyana initiative, a social and behaviour change (SBC) project led by Johns Hopkins Center for Communication Programs (CCP). This qualitative study serves to understand these behavioural components further from miners’, testers’, and other stakeholders’ perspectives. Eventually, these insights will help identify solutions to encourage miners to seek malaria testing and treatment services.

**Table 1:** Description of malaria testing and treatment available in public health facilities in Guyana

Malaria health service	Details
Testing brand	CareStart™
Treatment for *P. falciparum*	Day 1: 4 Coartem and 3 Primaquine tablets followed by 4 Coartem tablets 8 h after Day 2: 4 Coartem tablets at the same as the first dose, followed by 4 Coartem tablets every 12 h Day 3: 4 Coartem tablets every 12 h
Treatment for *P. vivax*	Day 1: 4 Chloroquine and 1 Primaquine tablets Day 2: 3 Chloroquine and 1 Primaquine tablets Day 3: 3 Chloroquine and 1 Primaquine tablets Day 4–14: 1 Primaquine tablet each day
Treatment for a mixed infection of *P. falciparum and p. vivax*	Day 1: 4 Coartem and 1 Primaquine tablets followed by 4 Coartem tablets 8 h after Day 2: 4 Coartem and 1 Primaquine tablets at the same time as the first dose, followed by 4 Coartem tablets every 12 h Day 3: 4 Coartem and 1 Primaquine tablets 12 h after followed by 4 Coartem tablets 12 h after Day 4–14: 1 Primaquine tablet daily at the same time as the first dose

## Methods

### Aims

This paper outlines results from a qualitative research study conducted to understand social, behavioural, and cultural reasons for gold miners’ care-seeking behaviours related to malaria testing and treatment in Regions 7 and 8 in Guyana. Camp managers, testers, miners, regional health staff, and regional leadership participated in semi-structured, in-depth interviews (IDIs) and focus group discussions (FGDs) to understand (1) how current testing and treatment services were made available to miners in remote communities in Regions 7 and 8, (2) how miners understood and used these services, and (3) what are associated challenges or opportunities related to the first two objectives.

### Guiding theories and definitions

Human-centered Design (HCD) is “a craft and discipline that applies a specific mindset and skillset to a creative problem-solving process, enabling the development of informed, sensitive, inclusive, purposeful, appealing, and innovative solutions” [35]. Tending to be more exploratory than hypothesis-driven, HCD draws upon anthropology, social science, ergonomics, human factors, and usability to prioritize end-user and stakeholder feedback as much as possible [36]. Although HCD has been packaged as an emerging discipline, qualitative research considers similar social and behavioural aspects through formative research, trials of improved practice, and behavioural trials.

All Breakthrough ACTION projects follow an SBC framework with three phases: (1) Define, (2) Design and Test, and (3) Apply. The SBC framework resembles the HCD process. First, the Define phase provides an opportunity to frame the nature of the problem with stakeholders’ input. For Guyana, in addition to qualitative research, the team conducted a literature review of malaria in the Guiana Shield. The Define phase culminates in “Insights” in which key findings are articulated and opportunities identified to set the direction for ideating and prototyping potential solutions in the Design and Test phase. Within the Design and Test phase, with the guidance of brainstorming questions from the “Insights” generated, partners, stakeholders, and miners co-design ideas to encourage miners to seek malaria testing and treatment. Solutions are further refined, prototyped, tested, iterated, and monitored, continuously improving the fidelity of the solutions compared to the previous iteration. Once these solutions are finalized, they will be implemented, monitored, evaluated, and scaled in the Apply phase.

SBC and HCD allow for a reframing of existing problems and much faster insight generation than traditional qualitative research. The SBC process does not necessarily uncover new insights that qualitative research cannot, but it can help reframe data in new ways. The HCD process offers the capacity for multiple team members, regardless of their familiarity with the health issue, to apply the same questioning eye. HCD does not disregard people’s expertise, but rather embraces it and recognizes that a more diverse team can produce richer data. HCD also provides a structured process to conduct qualitative research, develop solutions, and scale solutions for those unfamiliar with socio-behavioural research.

The Integrated Behaviour Model (IBM) is a conceptual model that helps identify factors that may influence behaviour [37]. This model builds on the Theory of Planned Behaviour [38], Social Cognitive Theory [39, 40], and theories of normative change [41, 42] to consider environmental factors—sociocultural factors, capacity to perform a behaviour, the salience of behaviour, structural/systems constraints, and habit/past performance—as well as individual beliefs and attitudes about behavioural options. For this study, three main behaviours were examined: (1) whether gold miners working in Regions 7 and 8 seek malaria testing within 24 h of the onset of a fever, (2) whether miners working in Region 7 and 8 seek MoPH-approved treatment and (3) if given MoPH-approved treatment, whether miners adhere to treatment completion. The following factors guided the analysis: capacity to perform the behaviour, the salience of the behaviour, structural and systems constraints, habit or past performance, overall attitudes toward the behaviours, and personal agency or efficacy [37]. In this study, the authors look specifically at care-seeking and non-care-seeking behavioural decisions to understand the factors that influence the above behaviours. Care seekers and non-care seekers are those who exhibit behaviours as exhibited in Table 2.

**Table 2:** Care seeker and non-care seeker definitions for malaria testing and treatment

	Care seekers	Non-care seekers
Testing	Care seekers are those who seek microscopy or RDTs to confirm whether they have malaria before any treatment begins, ideally within 24 h of the onset of a fever They can also use alternative treatments (non-medication) for immediate relief of symptoms, but still seek testing, are still defined as care seekers	Non-care seekers are those who do not seek microscopy or RDTs to confirm whether they have malaria, or miners who continually seek testing services for a desired outcome (e.g. continually testing themselves, hoping they get a positive test result to confirm their suspicion of malaria
Treatment	Care seekers are defined as those who do not self-treat with medication, first use MoPH-approved treatment courses, and adhere completely to the recommended treatment regimen Those who use additional alternative treatments (non-medication) for immediate relief of symptoms are still defined as care seekers	Non-care seekers are defined as those who only self-treat and do not use approved medication, or do not adhere to the medication prescribed to them from MoPH facilities. Self-treatment includes the use of bush medicine, home remedies, or over-the-counter access to malaria treatment medication. A miner who first self-treats with over-the-counter malaria medication, and then seeks MoPH treatment (after testing positive) first demonstrates non-treatment seeking behavior, followed by treatment-seeking behavior

**Fig. 1:**
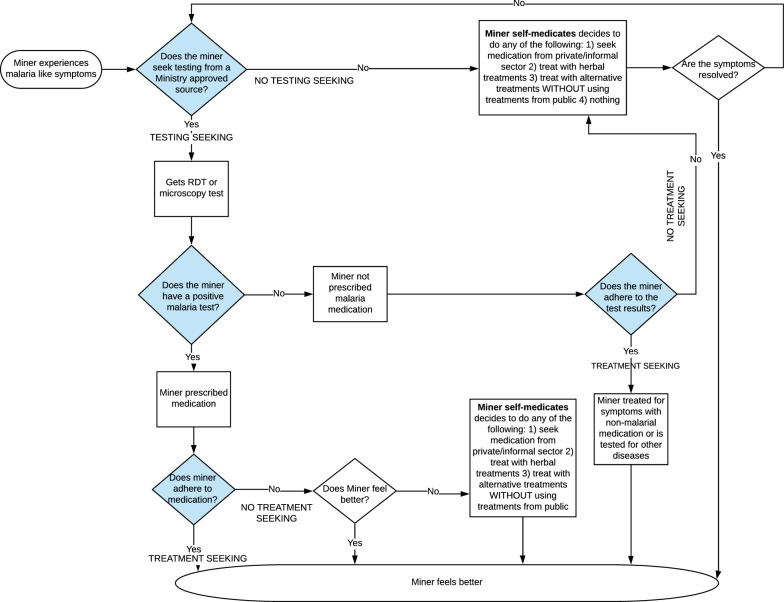
Journey map for malaria testing and treatment-seeking behaviour

In order to structure the decision-making analysis, the Breakthrough ACTION Guyana team first created a “journey map” that reflects the decision points that a miner may face in dealing with the experience of malaria-like symptoms [43, 44]. The journey map in Fig. 1 explains the moments in which miners could exhibit care-seeking and non-care seeking behaviour. An oval indicates start or endpoints, a rectangle indicates processes, and a blue diamond indicates the moments when miners make decisions about their care-seeking behaviour. This care-seeker or non-care seeker journey is based on whether miners seek testing within 24 h of feeling symptoms and adhere to the recommended treatment. When miners experience malaria-like symptoms and suspect they have malaria, they decide whether to seek malaria testing services (RDT or microscopy) at a public health service or not (first bifurcation of doer and non-doer behaviour). For those who do seek malaria testing services, if the test is positive, they are then prescribed medicines that are appropriate for the type of malaria they have. If miners adhere to the treatment, they demonstrate doer treatment behaviour. If they do not adhere to medication, they demonstrate non-doer behaviour. Miners who use non-medication, alternative treatments (e.g., home remedies) and are still considered doers so long as they seek and adhere to approved treatment in addition to the alternative or herbal remedies.

For those who do not seek malaria testing services from a public health service, they make a choice to do any of the following, either alone or in combination: (1) seek medication from a private or informal sector facility, (2) use herbal treatments like quinine tea, or (3) use alternative treatments (e.g., coconut water or disinfectant solution) that are not captured in the first two options, or (4) nothing at all. If they use any of these four options and do not take any medication from MoPH-approved sources, the miner demonstrates non-treatment seeking behaviour. If miners do not feel better, they may consider to test for malaria for the time.

### Ethical review

The Johns Hopkins University School of Public Health Institutional Review Board classified this activity as a non-research activity and within the domain of public health practice. Additionally, the Ministry of Public Health, Guyana gave ethical approval. All participants underwent an oral consent process, which researchers confirmed through signed documentation.

### Research site and recruitment

The selection of mining camps for interviews was purposive. Mining camps were selected if they were geographically accessible and close to Puruni (Region 7) and Mahdia (Region 8) and had a RDT tester trained nearby. In Region 7, the team was based out of Puruni, a landing of fewer than 1000 people. In Region 8, the team was based out of the administrative capital Mahdia, with a population fewer than 4000 people. Travelling within the interior of Guyana requires trucks that can manage unpaved rocky terrain. Due to these constraints, only mining camps located within three hours of Puruni and Mahdia were selected. Selected camps represented a range of large, medium, and small operations with different degrees of professionalism and organizational infrastructure. Before research activities began, members of the research team oriented and sought permission from mining camp managers during a preparatory field visit. Miners within participating mining camps did not participate if they were not interested.

Before to fieldwork, two teams of eight researchers each consisting of experienced social scientists, MoPH field workers, and design specialists, trained in Georgetown on the techniques of research, the use of the research guides (lines of inquiry), and research ethics. From October 26 to November 1, 2018, the research team conducted IDIs and FGDs, each of which lasted up to 60 min. Ultimately, each interviewer decided which questions to ask and how to probe. For the miners, conversation topics included personal background and working context; perceived health risks, especially related to malaria; knowledge of malaria; and behaviours and perceptions related to malaria testing, treatment, and prevention. For other stakeholders, conversation topics included their experience and perspectives of malaria within gold mining camps. Semi-structured IDIs were conducted in teams of two or three: a facilitator who led the interview; and one to two researchers who took notes and managed the recorder. At least one researcher who could speak Guyanese Creole was included on each interview team to provide balanced representation and interpretation if needed. For FGDs, a similar facilitator structure and lines of inquiry were used. FGDs can offer insights on community norms and allow for exploration of disagreements among participants that are not possible with in-depth interviews. IDIs and FGDs were conducted in a convenient, confidential location for participants within the mining camps. Due to the language limitations of the research team, only miners who could speak Guyanese Creole or English participated (Portuguese and Spanish speaking miners were available).

The research team recruited miners through a convenience and variance-based sampling method and a care-seeking/non-care seeking analysis was applied retroactively to the data. The research team sought to interview miners who exhibited a variety of experiences with malaria testing and treatment. Inclusion criteria included miners who:

- Had and had not been tested for malaria
- Did and did not have access to RDTs at camps
- Had and had not tested for malaria or received a malaria diagnosis
- Had adhered to or defaulted on treatment
- Had sought malaria treatment quickly and those who delayed treatment
- Had sought malaria treatment from informal and informal sources
- Guyanese and non-Guyanese workers

All participants were verbally consented based on information about the nature of the study, the rights of participants (e.g., confidentiality and the voluntary nature of their participation), and the use of recording equipment. Researchers signed consent forms to confirm they had gone through the consenting process with each participant.

### Data analysis

The research team used a framework approach to analyze the data, based on Gale et al. [45]. First, one researcher (SY) listened and transcribed key quotes from each recorded interview if they pertained to the research objectives. After listening to all of the interviews, a list of key themes and traits for testing and treatment-seeking and non-seeking behaviour (e.g., access to care, trust in public hospital system, adherence challenges) was developed and entered into a spreadsheet. The same researcher (SY) then organized quotes from each interview into the spreadsheet, and each interview was marked as care-seeking or non-care seeking, based on supporting quotations. Participants could demonstrate both care-seeker and non-care seeker behaviour, because behaviours and attitudes may have changed throughout their mining experience. An overview tab summarized which interviews exhibited which care seeker and non-care seeker behaviour, followed by data from each campsite. An additional member of the research team (JO) reviewed and helped clarify emerging themes. Any conflicts in coding and organization of the data were reviewed, discussed, and resolved between SY and JO.

## Results

As outlined in Table 3, a total of 109 individuals were interviewed across 58 recordings, including 70 miners, 11 of which participated in FGDs in two recordings, and the rest in individual IDIs. Seventeen camp managers, 10 RDT testers (including those who served as other roles), seven pharmacists and community health workers (CHWs), and nine other individuals participated. The “other” category includes local administrative officials including the regional executive officer, regional health officer, regional chairman, monitoring and evaluation officers, or other hospital staff. One of the recordings from Region 8 was compromised due to the quality of the sound and researcher error in operating the recording equipment. Sixteen miners exhibited past or current testing seeking behaviour; seven miners had no past or current testing seeking behaviour; 16 miners had past or current treatment-seeking behaviour; and 12 miners had no past or current treatment-seeking behaviour. In the FGDs, it was not possible to determine how many miners demonstrated testing and treatment seeking behaviours from the recording alone. As a result, they were not included in the above totals, but their responses are analysed. Major themes are organized by miners’ decision to seek testing; their decision of which treatment options to pursue, use of alternative treatment options, and treatment adherence. Data saturation was achieved among miners, camp owners/managers, and testers; not enough of the other stakeholders were interviewed to achieve saturation.

**Table 3:** Summary of participant profiles by region

	Location	Miners	Owners/managers	RDT testers	Pharmacists and CHWs	Other^a^	Total
Region 7	Puruni (across river) camp	5	2				7
	Little Soiree Camps		4				4
	Bacchus Camp	7	2				9
	Tiger Creek			1			1
	Mikey’s Camp	8	2				10
	Kumang Kumang			1			1
	Takatu	8	2	1(1)^b^			11
	Puruni (Landing)	3		2	2		7
	Bartica				2	2	4
	*TOTAL*	*31*	*12*	*5*	*4*	*2*	*54*
Region 8	Sala Bora Camp	7	1				8
	Minnehaha Camp	5	1	1			7
	Eagle Mountain Camp	9	1				10
	Tussurrow Camp	9	1				10
	Mikobi Health Post			(1)^b^	1		1
	R&V Camp	9	1	(1)^b^			10
	Madhia			(1)^b^	2	7	9
	Total	39	5	1	3	7	55

^a^Other includes regional executive officer, regional health officer, regional chairman, monitoring and evaluation officers, or other hospital staff

^b^These individuals served multiple roles (e.g. camp manager and tester). Their dual positions are rerecorded but not double-counted into the totals

### Decision to seek malaria testing services

#### Testing seeking: knowing for sure

Malaria is a prevalent and very well-known issue in the interior, and miners know the symptoms well. Malaria is strongly associated with being in the interior; such that *“basically, almost everywhere in Guyana, once you work in the interior, malaria is probable”* (Eagle Mountain Mining Camp, Region 8). According to a miner in Minehaha, Region 8: *“Nobody can prevent malaria. It will come. When you think you get malaria, you get malaria.”* Given the prevalence of malaria in these regions, its symptoms are well understood. Participants could articulate fevers, cold sweats, body pain, and other symptoms they experienced when they had malaria. For example, a miner from R&V Mining Camp in Region 8 reported the following symptoms when he last had malaria:

“*Got a strong, strong fever, and yet, you feel cold, and you know your body hot when you touch the skin. But then when any little breeze blow, you cold cold cold. You cover up with your sheet, and it keeps cold. Then your body is always in pain.”*

About half of the participants used testing services (microscopy or RDT) previously, but only a handful had consistently used it every time they suspected they had malaria. Among miners who did seek malaria testing services, knowledge of the type of malaria one had ensured they would be treated for the right strain. A miner from Sala Bora in Region 8 shared the following advice with his peers:

*“I tell them it’s best to go to the hospital and take a smear, stop buying the tablet, it could still be in your system. Taking the tablet could keep it down in your system, it hiding in your liver or somewhere until it decide to take over you again.”*

For other miners who are more confident they have malaria, testing assured distinguishing which malaria miners had and that the appropriate treatment could be used:

*“Normally, because you know it’s malaria, because of the symptoms, you take the tablets from the shop. We know the feelings, everybody know the feelings, that’s malaria. So after the testing came, you know directly what type you got”* (R&V Mining Camp, Region 8).

Finally, some miners used malaria tests to confirm what they are already feeling:

“*From time to time when I take tests, depend on how I feel and the type of malaria, I would determine which is which. I would check on my joints, my back, the different symptoms”* (R&V Mining Camp, Region 8).

Testing services also provide a strong sense of relief for miners to know why one is sick and that they will be able to receive medication to relieve their symptoms. As a tester in Takatu, Region 7 recounts:

“*[Miners] want to know if they medication here too. When they get a test, and it show positive, they feel happy cause, you know they gon feel good the next day.*” Simultaneously, however, malaria tests can also be frustrating for miners who receive a negative result but still have painful symptoms.

The convenience of RDT services was a benefit. As described by several miners, there is a long-standing tendency to self-diagnose and self-treat to manage one’s symptoms in the interior. They only resort to testing and treatment at public health services when the pain becomes serious enough. Distance from health facilities is a well-understood challenge within the Guyana hinterland, as reflected in accounts pre-dating the availability of RDT services:

*“That time when I had malaria, [I] used to take Artecom [malaria drug], depends if you feel you know the treatment, you have the symptoms, take Artecom. That time I didn’t take a smear because the interior was far, no hospital.”*

The lack of access encouraged miners to develop workarounds and access treatment when they could:

“*Normally the boss used to bring in the treatments, but those treatments are expensive. So, they used to get, I can’t remember the name. But we normally would have had treatment, but then you never used to do any tests. You just know this is malaria feelings, you buy a packet and you drink it”* (R&V Mining Camp, Camp Manager, Region 8).

The presence of the RDT testing services has made it easier, because, as a miner reported,

*“now we get the testing and treatment right up here. We ain’t gotta go far. We get the treatment, the tablets. More easier”* (Eagle Mountain, Region 8).

A camp manager from Sala Bora Mining Camp, Region 8 believes the RDT testers is a time-saver:

*“Rather than wasting time when you go out there, and it’s not malaria, if you get a rapid test there, you do it, then you know it’s not malaria, then I would ask for panadol (pain reliever).”*

This benefit of saving time was not necessarily consistent across all experiences. While there are existing resources to treat malaria, RDT testers do not have resources for other diseases:

*“I enjoy the most, when I find the patient positive, I feel good because I can give them treatment. If they are negative, I feel kind of worried. Sometimes the symptoms they telling you they getting, it’s a worry. What else could this be?”* (Tester, Puruni Landing, Region 7).

As a result, miners ultimately still have to deal with painful symptoms from other diseases if they don’t have malaria:

*“If someone came with the case of malaria, it would show up negative on the tests… and I aint got no means. Y’all would look after dengue too? Because dengue in here a lot*” (Tester, Kumang Kumang, Region 7).

A pharmacist in Mahdia, Region 8 who provides RDTs (though not approved by MoPH) recounted a situation in which the RDTs caused a delay in care and treatment:

*“I think yesterday there is a situation where two persons, a girl and her husband. They did a test but it was negative, they continued to say that he knows he has malaria, say he demand he does another. I did for both for them, and they were positive, and I send them back to the hospital, and he came back with his treatment. I think his wife was annoyed maybe because of the procedure, she did not redo the test, but he did, so he got his treatment.”*

This incident not only speaks to the frustration of delayed care and treatment but also the potential unreliability of non-MoPH approved RDTs.

#### Non-testing seeking: confidence in self-diagnosis or lack of awareness

Seven miners demonstrated clear non-care seeker behaviour. Miners who chose not to seek testing services described, with confidence, they could self-diagnose:

*“Basically you get different symptoms. Vivax, sometimes you would get diarrhea. For me I would know. Falciparum, my joints along with the back pain, inside fever, headache, my joints, that is how I would use to know”* (R&V Mining Camp, Region 8).

They felt that their experience with malaria helped them determine what type of malaria they had, and how they should treat it:

“*By the time someone feels the way with the symptoms, they actually know it’s malaria, so they take treatment”* (Kumang Kumang Mining Camp, Region 7).

For miners who use tests to affirm their self-diagnosis, they continue to test until they get a positive result:

*“Some persons I know have malaria, so they will be back and forth until they get the results they’re looking for”* (Pharmacist, Mahdia, Region 8).

Otherwise, miners would use testing services at public health services after their self-treatment regimens had failed because they ultimately wanted access to MoPH-treatment.

Low use of malaria testing services among miners may also be due to a low understanding and awareness that RDT services are available. The majority of miners interviewed were not aware that there were testers in or close by to their camp. Part of this lack of awareness was due to the absence and movement of testers themselves, who may temporarily or permanently leave their postings (of the 16 locations, 8 locations were supposed to have a designated tester, of which only about 4–5 were present). In some cases, news of a tester quickly spread among the miners: *“After a few months, everyone aware the corner shop is one of the main location that would do malaria testing, and they would normally ask somebody where those malaria testers are”* (Tester, Puruni Landing, Region 7).

Some miners overuse testers for their health resources. A tester in Takatu, Region 7 mentioned a miner who sought malaria testing, though he had no symptoms:

“*If they come and say, oh you’re doing malaria testing. Come give me a test. You have to explain to them… no I can’t waste this thing. Sometimes I get the last test, and I do it on you, and because me and you is friends, and someone come really in need of the test, and you can’t do it… He come, and he said I want to do the test. He ain’t got no fever, or headache. And then he said oh a couple of days.”*

### Treatment decisions

Miners either (1) seek treatment from MoPH-approved sources (e.g., from trained testers or public health facilities), (2) self-treat and seek medication from non-MoPH-approved sources other (e.g., pharmacist, camp managers, or shops), (3) self-treat with alternative options (e.g., herbs and non-medication options), or (4) do nothing. Throughout the interviews, miners stated that they did not consistently do the same behaviour each time they experienced malaria-like symptoms and that they often self-treated with alternative options that were combined with either the first or second option described above. Regarding adherence, miners varied due to the relief of malaria symptoms and negative side effects.

#### Treatment seekers: reliable results

Several participants spoke about the trustworthiness of malaria treatment services provided from public services, which were perceived as a place that gives the correct treatment:

“*Remember we got testing, and people telling you the right treatment is the hospital treatment. But the treatment from the drug store, or the private doctor, start fallin’ into the symptoms. Cause every time I go to the hospital, I get the right tablet I feel better. I go to a private doctor, they give me a fancy tablet, but nothing really to [help me]”* (Eagle Mountain, Region 8).

Additionally, another miner who had experienced poor health outcomes from self-treating with medication from private or informal providers developed an affinity for public hospitals:

“*In the old days, I try the self*- *treatment thing but it never work, and then I started believing in the hospital” (Eagle Mountain, Region 8).*

Trust of public medications may have come from the belief that they work better than other medications: *“I never buy the drugs outside, I prefer the hospital drugs, because I feel those work more better” (*Eagle Mountain, Region 8).

Public health systems are not without challenges though. One miner did state that private hospitals were better for the public, given the speed with which private hospitals could see patients:

“*I would say they would give you more attention. Public most people might rush to because they might not have the finance. Suppose you don’t have a finance concern, you’d get fast treatment at the[private] hospital”* (Tussurrow, Region 8).

#### Non-treatment seeking: no other option and symptom treating

In most situations, miners knew that they should seek treatment from public health services and complete the treatment. However, miners often didn’t because there were no treatment services close by or transportation choices were not viable. As one miner stated, not taking the recommended treatment was a function of how far they are from services:

“*Well, there was no access to get treatment on this side. Buy tablet, take the tablet (may not be the right tablet or the right amount) to ease down the malaria next couple of days”* (Eagle Mountain Mining Camp, Region 8).

Miners who used alternative treatment options demonstrated their resourcefulness in a constrained environment. While in the back dam (interior, rural, and/or undeveloped areas and locations where mining is conducted) transportation to seek testing was challenging, and the lack of it encouraged the miners to have medication on hand:

*“That time when you go in the back dam you walk with some tablet, because not like here now, that time the transportation hard, and you got to pay money to go out”* (R&V Mining Camp, Region 8).

Another miner from the R&V Mining Camp mentioned that his wife would take malaria pills from a malaria specialist so he would have the treatment with him before testers were trained in his camp:

“*But my wife, she works at hospital. So she would get treatment for me. She would go to the malaria specialist. She would get treatment for both malarias. She would bring it in, send it in to me”* (R&V Mining Camp, Region 8).

Due to work pressure, miners are generally focused on solutions to manage their pain and general symptoms. Miners spoke about the tendency in their community to work through pain to not forgo a day’s wage. A miner in Tussurrow, Region 8, reported,

*“Sometime you see a man trembling, he don’t wanna go home, because money he make. He trying to drink tablets to keep it down until he make the money to go home… Sometimes he can’t make it no more and he go home, because he just vomiting.”*

Every missed day is a day of lost income, whether it is due to feeling unwell, efforts to seek testing services or the side effects of the treatment. Camp managers may also contribute toward the pressure of work:

“*From the little I know, you may have a thing going into a camp area, we call the general manager may not want to stop working for the men to do the smear. Because all they think about is production and productivity. Having down time means so much to them, but they aren’t looking at the worker’s health at the same time”* (Regional Health Officer, Bartica, Region 7).

In addition to individual miners feeling the economic pressures of the mining industry, there are wider community implications as well. The Regional Executive Officer in Bartica, Region 7 sums up the larger role that the mining industry plays for the region:

“*Our economy depends on mining, and if we don’t have a healthy workforce, even from the miner perspective, if all our men got affected came down with malaria, then production is affected. Then our region as a whole is affected. We are depending on [the] whole on the mining sector. If the mining sector does well, we up and well.”*

Miners choose to forgo testing and prefer treatment options first so they can return to work as soon as possible.

The way that malaria is treated is not consistent across miners. Some miners understand that if they do not fully take all of the treatment, or do not get tested to identify the type of malaria that needs to be treated, they risk getting sicker. But other miners attempt to manage their symptoms as much as possible on their own.

### Alternative and herbal treatments

Several participants mentioned following non-traditional treatments that they had used to resolve malaria symptoms. It should be noted that it is unclear, based on responses, whether participants thought that these methods alleviated the symptoms or whether it stopped the malaria entirely. The following items had been used by miners previously to relieve their malaria symptoms: coconut water, Jase fluid (disinfectant), Guinness beer and mustard, orange juice, Panadol (paracetamol), and pain killers. These alternative treatments were either recommended by peers or informed by miners’ understanding of treatment. For example, one participant who consumed 12 bottles of orange juice when he was experiencing malaria symptoms did so because he had heard that Vitamin C is good for people who are in poor health:

*First time I had malaria I feel like the world coming to an end. Feelings. I didn’t have treatment where I was, I drink like 12 Guzzler (orange juice). I think within the same day, I could have worked back. From the back dam, straight up to the junction, waiting on transportation at junction, it didn’t come. I was just, the best shot is to have orange juice. When your immune system down, gotta get Vitamin C. And orange juice have vitamin C. (Tussurrow, Region 8)*

Miners identified alternative treatments as reagents that could raise or lower malaria symptom intensity, thereby affecting the test results:

“*I know things like drinking Panadol, ibuprofen, antibiotics, those things will hide it… Yeah I experienced it. Drink the Panadol it hide it. Coconut water, hide it [from the test]”* (Tussurrow, Region 8).

Other things, such as beer, were believed to make malaria more pronounced:

*What I do, and it works, if you drink a beer, something, you know it raise the parasite. Actually get the full symptoms. Raise the symptoms, you know it is malaria. The first time I had it, that is what I do. The symptoms, they went tricking, and a friend said you should drink a beer. And after then inside the bush, you drink a beer, and the beer help the parasite produce, you know this is malaria. It show in your test.* (Miner, Tussurrow, Region 8)

Miners also mentioned herbal treatments to prevent and protect themselves against malaria: “*Sometimes I use bitters, so it can’t really get into the blood. We use most bitters. Like karela bush or something like that”* (R&V Mining Camp, Region 8).

For the most part, miners used alternative and herbal treatments either on their own or in combination with medicines from public, private, or informal sources. The use of these treatments was likely due to recommendations from peers and the observational efficacy that they worked because they temporarily reduced the symptoms.

#### Adherence

Commonly referred to as “drinking out,” adherence to medication is also challenging for malaria control among the miner population. Those who did not complete their treatment understood they would not eliminate malaria, but only address the symptoms and could relapse back into malaria:

*The thing is, you gotta make sure you don’t miss your treatment. Because if you miss your treatment then you can get malaria. [The treatment] would only be active in your system for a week. Being able to fight off the virus for the week, after that you need to take the treatment.* (R&V Mining Camp, Region 8)

Miners who completed the treatment understood that if they wanted to feel better, they would need to adhere to their treatment in full. For others, advice from the medical community motivated them to finish their treatment:

*“I drink it because they tell me to drink it to feel good. Can’t short my treatment”* (Sala Bora Mining Camp, Region 8).

It was well known that miners do not adhere to prescribed malaria treatment. Miners cited side effects, such as itching, the taste of the medication, and drowsiness, as a reason why they stopped their treatment:

*“[The medication has] bad taste, but it would be on your taste buds in your glands for a few minutes, maybe an hour or so”* (Minehaha Mining Camp, Region 8).

The course of the treatment itself often exacerbated symptoms first, before relieving them. One miner stated the treatment “*chops your body*” (Tussurrow, Region 8). It was typically at this point, when miners felt the worst, or right after when they felt better, that they would stop taking treatment. Those who accepted this as a normal part of the treatment course adhered:

*“No it’s the normal thing, when you take the treatment, it more worse, you feel worse before you feel better”* (Tussurrow, Region 8).

However, miners who thought this was not considered normal interpreted feeling worse as evidence that treatment did not work for them. Miners also identified the improvement of symptoms as a sign that treatment was working and that it was acceptable to stop. When asked why he stopped taking the medication, a miner from Kumang Kumang Mining Camp, Region 7, stated.

“*Cause I don’t like the tablets, and from the time I start feeling good, I just stop drink it.”*

Even with the presence of RDTs and treatment at the mining camps, miners did not finish their treatment:

*“If I get malaria presently, I will call [the camp manager], because he is the manager. He has the test. If it’s malaria or what type of malaria you know. If it’s positive we will stay [at the camp], but drink the treatment…Well first, when as soon as I get good, one or two day, sometime I stop. Before I used to drink it out clean. But now, when I feel good, I stop*” (R&V Mining Camp, Region 8).

No miner mentioned that they did not finish taking the treatment to save it for later. A miner from Kumang Kumang, Region 7, threw away her leftover treatment because she thought it would not be useful the next time:

*“If I don’t drink out the treatment, I throw out the rest. Because it’s not all the time you would get the same malaria. Right? So the same treatment don’t work with the same malaria.”*

Less explicitly discussed were scenarios when miners tested negative for malaria, but still sought out treatment. One miner from Sala Bora, Region 8 discussed an instance in which he got a negative malaria test result, but still sought Artefan treatment (also known as Coartem and contains 80 mg of artemether and 480 mg of lumefantrine) [46].

Camp managers sometimes played a supportive role to ensure that their workers adhered to the medication regimen:

*“The testing, when a worker tells me they’re not feeling well, well I would ask questions, if they headaches, fever, I would ask how long. I would say ok, I would prepare, I would ask them to come off, and I would take them out myself, or my other half would take them to the hospital to get tested and medication, and bring them in, and take them out and bring them in. I would set my alarm whether on my phone or the clock. I would give them the dosage myself, and set and alarm so they don’t over drink, or before or after the time. Give them on the stop. I hold onto the medication”* (Camp Manager, Sala Bora, Region 8).

Table 4 summarizes research findings by different IBM constructs. The majority of the environmental and individual factors are categorized in the structural/system constraints, habit/past performance, and overall attitudes.

**Table 4:** Summary of testing and treatment examples for components of the Integrated Behaviour Model

IBM model constructs	Testing seeking	No testing seeking	Treatment seeking	No treatment seeking
Environmental factors				
Capacity to perform the behaviour	Knowledge and awareness of RDT services	Confidence in experience with malaria to self-diagnose	Aware of importance to adherence to correct treatment	Inability to manage pain before reaching public health facilities
Salience of behaviour	Knowledge that knowing malaria strain is important	Desire to seek treatment as soon as possible	Desire to fully treat malaria from their body	Unable to manage pain until possible to reach public health facility
Structural/systems constraints	Geographic proximity of RDT services	Easy access to malaria treatment without the prerequisite of testing services	Speed and ease of private health facility	Access challenges to facilities (before RDT program in place) (e.g., transportation, distance) Easy access to malaria medication and alternative treatments Desire to return to work as soon as possible
Habit/past performance	Poor past experiences with self-treatment from private or informal health facilities (empirical efficacy)	Confidence in experience with malaria to self-diagnose (empirical efficacy)	Miner’s self-treatment did not work (empirical efficacy)	Confidence in experience with malaria to know which type they have to self-treat or that non-adherence works (empirical efficacy)
Individual factors				
Overall attitude	Assurance to know which malaria strain miner has Ensure they get treated for the right illness before taking treatment	Not important to seek malaria testing	Trustworthiness of treatment from public health system	Adherence: Recommendation from medical treatment Personal preference for alternative treatments to deal with the pain
Personal agency	Ability to seek testing from public health facilities	Ability to self-identify strain of malaria	Ability to seek treatment from public health facilities	Ability to find an alternative medication or medication from non-public health facilities

## Discussion

This study provides further insights into the perceived value of malaria testing and reasons why miners may not pursue the MoPH-recommended course of treatment in Regions 7 and 8. As observed in a study among gold miners in French Guiana, treatment adherence rates were higher among miners who reported that they had been tested, implying that improving testing rates is a critical upstream factor [47]. The tendency to self-treat aligns with other studies throughout the Guiana Shield, specifically in French Guiana and self-medication of an artemisinin-based combination [9, 10, 48]. Results from this study around the pressures to return to work are consistent with the results from a study in Guyana and Suriname: miners self-medicate so they can resume mining activities as soon as possible and are willing to pay what is required to address their symptoms [49]. The tendency of miners to trust public health institutions contributes to an existing body of research documenting the same trend across other health areas [50, 51]. Access to health services in remote regions is a well-known challenge throughout the Guiana Shield especially in the interior but is not well-documented for Regions 7 and 8 among mining communities. Study participants across regions reported that a variety of treatments, ranging from MoPH-approved medication, unregulated treatments, and alternative methods, were available in varying quality. This aligns with a previous study of anti-malarials available in Guyana and Suriname, which found that more anti-malarials from the private sector failed quality-control tests than those from the informal sector, although that private sector facilities are licensed facilities and informal sector facilities are unlicensed [49].

The results of this study showed a need for programmes to increase visibility, trustworthiness, and demand for the RDT testers. Due to the side effects of treatment and the financial pressure to return to work, most miners stopped treatment as soon as they felt better. Financial incentives or improved company sick-leave policies could encourage miners to seek the recommended testing and treatment, and complete the treatment rather than take shortcuts to quickly address symptoms.

There is scope for multiple stakeholders to collaborate and target miners, and migrant and mobile populations in general. Research from Nepal and Cambodia suggests that targeted interventions for migrant and mobile populations will be important given these populations are at the highest risk of receiving and passing on malaria [52, 53]. Based on a literature review of malaria interventions for migrant and mobile populations, intersectoral collaboration can lead to increased malaria knowledge and access to malaria testing and treatment services and recommends that specific behaviour change communication for migrant populations [54]. For example, in Indonesia, mining companies implemented and supported malaria control services for its workers and local population, which is linked to a decrease in malaria cases for miners and local communities [55]. Within Southeast Asia, promising interventions include intercepting populations on their travel route, enlisting fellow migrants or remote villagers as volunteer migrant malaria workers, developing mobile clinics, setting up malaria posts in high traffic areas (such as border crossings), and working with employers to provide testing and treatment services [56, 57]. Finally, while the practice of self-diagnosis and treatment is not ideal, programs may need to incorporate these tendencies into interventions, as demonstrated by “Malakit” in French Guiana. “Malakits” include RDTs, appropriate and complete malaria treatment for *P. falciparum* and *P. vivax*, alternative medication if RDT results are negative, and visual instructions for miners on how to use the kit when they suspect they may have malaria [48]. While this is not a “gold standard” approach, in which trained public health officials monitor malaria testing and treatment, it offers a tailored approach to longstanding care-seeking behaviours among miners.

There are several limitations to this study. Given the nature of the sample size, purposive sampling method, and location of research activities, these interviews are not representative of all the regions, of gold mining populations, nor of all interior populations in Guyana. Study site locations were limited by how easily the research team could access transportation to mining camps. As a result, the experiences for communities even further in the hinterlands may not be fully captured. Due to language limitations, perspectives from Brazilian and Venezuelan miners were not included as only miners who could speak English and Guyanese Creole were interviewed.

## Conclusion

Malaria is an enduring public health issue in Guyana and an almost universal experience among Region 7 and 8 gold miners. Miners who sought testing and treatment demonstrated a preference for public hospital services and the belief that testing would be valuable for the correct diagnosis and appropriate treatment. Miners who did not seek testing and treatment services were limited by distances, cost, and time associated with leaving work to seek services. They also faced limited access to government-approved services and products, over-confidence in their ability to self-diagnose based on experience, perceived efficacy of non-official treatments, and difficulty in dealing with treatment side effects. Future programs and interventions should consider ways to overcome these factors to improve gold miner use of MoPH-approved malaria services and products.

## Data Availability

Data sharing is not applicable to this article as no datasets were generated or analyzed during the current study.

## Abbreviations

CCP: Center for Communication Programs
CHW: Community Health Worker
FGD: Focus Group Discussion
HCD: Human-centered design
IEC: Information, education and communication
IBM: Integrated behaviour model
IDI: In-depth interview
LLIN: Long-lasting insecticide-treated net
MoPH: Ministry of Public Health
NMP: National Malaria Programme
PAHO: Pan American Health Organization
RDT: Rapid diagnostic test
SBC: Social behaviour change
WHO: World Health Organization
